# Morphology, Multilocus Phylogeny, and Toxin Analysis Reveal *Amanita albolimbata*, the First Lethal *Amanita* Species From Benin, West Africa

**DOI:** 10.3389/fmicb.2020.599047

**Published:** 2020-11-20

**Authors:** Jean Evans I. Codjia, Qing Cai, Sheng Wen Zhou, Hong Luo, Martin Ryberg, Nourou S. Yorou, Zhu L. Yang

**Affiliations:** ^1^CAS Key Laboratory for Plant Diversity and Biogeography of East Asia, Kunming Institute of Botany, Chinese Academy of Sciences, Kunming, China; ^2^University of Chinese Academy of Sciences, Beijing, China; ^3^Research Unit Tropical Mycology and Plant-Soil Fungi Interactions, Faculty of Agronomy, University of Parakou, Parakou, Benin; ^4^Department of Organismal Biology, Uppsala University, Uppsala, Sweden

**Keywords:** amatoxins, phallotoxins, poisoning risk, taxonomy, tropical Africa

## Abstract

Many species of *Amanita* sect. *Phalloideae* (Fr.) Quél. cause death of people after consumption around the world. *Amanita albolimbata*, a new species of *A.* sect. *Phalloideae* from Benin, is described here. The taxon represents the first lethal species of *A.* sect. *Phalloideae* known from Benin. Morphology and molecular phylogenetic analyses based on five genes (ITS, nrLSU, *rpb2*, *tef1-*α, and β*-tubulin*) revealed that *A. albolimbata* is a distinct species. The species is characterized by its smooth, white pileus sometimes covered by a patchy volval remnant, a bulbous stipe with a white limbate volva, broadly ellipsoid to ellipsoid, amyloid basidiospores, and abundant inflated cells in the volva. Screening for the most notorious toxins by liquid chromatography–high-resolution mass spectrometry revealed the presence of α-amanitin, β-amanitin, and phallacidin in *A. albolimbata*.

## Introduction

Some species of *Amanita* Pers. are edible, whereas others are poisonous. All lethally poisonous *Amanita* species are in *A.* sect. *Phalloideae* that is characterized by a non-striate and non-appendiculate pileus and attenuate lamellulae, the presence of persistent annulus, a limbate volva on the bulbous stipe base, and amyloid basidiospores (Corner and Bas, 1962; Bas, 1969; Tulloss and Bhandary, 1992; Yang, 1997, 2005, 2015). Members of the section are responsible for greater than 90% of mushroom fatalities reported worldwide (Bresinsky and Besl, 1990; Unluoglu and Tayfur, 2003; Cai et al., 2014, 2016; Li et al., 2015). In Central Europe and North America, lethal amanitas such as *A. bisporigera* G.F. Atk., *A. phalloides* (Vaill.: Fr.) Link, *A. suballiacea* (Murrill) Murrill, *A. verna* (Bull.) Lam., and *A. virosa* Bertill. have caused dramatic human and animal poisoning cases (Wieland, 1973, 1986; Bresinsky and Besl, 1985). It has been reported that *A. exitialis* Zhu L. Yang and T. H. Li, *A. fuliginea* Hongo, *A. fuligineoides* P. Zhang and Zhu L. Yang, *A. pallidorosea* P. Zhang and Zhu L. Yang, *A. rimosa* P. Zhang and Zhu L. Yang, and *A. subjunquillea* S. Imai cause a lot of fungal poisoning cases in East Asia (Zhang et al., 2010; Deng et al., 2011; Chen et al., 2014; Li et al., 2014, 2020; Cai et al., 2016). The toxins in lethal amanitas are mainly amatoxins, phallotoxins, and virotoxins (Wieland et al., 1983; Wieland, 1986; Cai et al., 2016). Among them, amatoxins are 10–20 times more toxic than the other ones and represent the major toxins responsible for human poisoning (Li and Oberlies, 2005). These cyclopeptide toxins are able to resist high temperatures, and their consumption can cause severe liver and renal failure (Wieland, 1973, 1986; Chen et al., 2014).

Lethal amanitas have been extensively studied in Asia, Europe, and America, where more than 50 toxic taxa have been described (Zhang et al., 2010; Cai et al., 2014, 2016; Li et al., 2015; Yang, 2015; Thongbai et al., 2017; Cui et al., 2018). Although the genus *Amanita* is known worldwide, few taxa of the genus have been reported from tropical Africa (Walleyn and Verbeken, 1998; Eyi-Ndong et al., 2011; Yorou et al., 2014; Härkönen et al., 2015; De Kesel et al., 2017; Fraiture et al., 2019; Tulloss et al., 2020). Six species belonging to *A.* sect. *Phalloideae* are known from tropical Africa, of which three, including *A. alliodora* Pat., *A. murinacea* Pat., and *A. thejoleuca* Pat., were described from Madagascar (Patrouillard, 1924; Fraiture et al., 2019). The other three species, *A. bweyeyensis* Fraiture, Raspé and Degreef, *A. harkoneniana* Fraiture and Saarimäki, and *A. strophiolata* Beeli were described from DR Congo (Beeli, 1927, 1935; Fraiture et al., 2019).

In this study, a new member of *A.* sect. *Phalloideae* from tropical West Africa is described. Its macromorphological and micromorphological characteristics, as well as its phylogenetic relationships with other *Amanita* species are discussed. In addition, the screening of the species for the known toxins occurring in *Amanita* is reported.

## Materials and Methods

### Collections and Preservation

**FIGURE 1:**
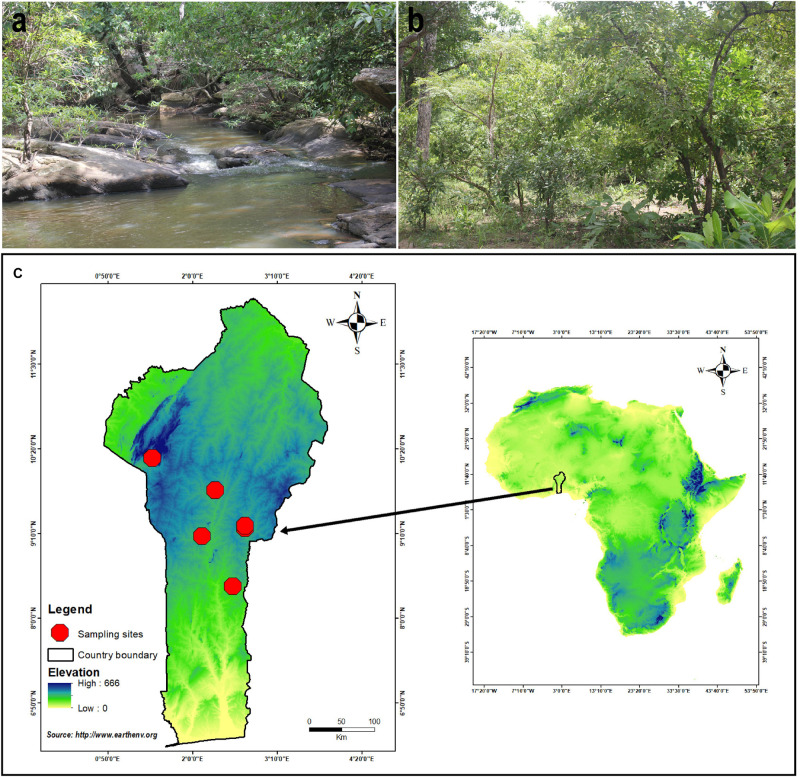
Distribution of *A. albolimbata* J.E.I. Codjia, N.S. Yorou and Zhu L. Yang: **(a)** Gallery forest associated with *Uapaca guineensis*. **(b)** Woodland associated with *Uapaca togoensis* and *Isoberlinia doka*. **(c)** Location of the sampling sites.

Specimens were opportunistically collected in Benin, West Africa (Figure 1), during the rainy season from June to September (2018-2019), especially in the forest dominated by Fabaceae/Leguminosae (*Isoberlinia* Craib and Stapf ex Holland, *Anthonotha* P. Beauv., *Berlinia* Sol. ex Hook. f.), Phyllanthaceae (*Uapaca* Baill.), and Dipterocarpaceae (*Monotes* A. DC.). Specimens were photographed *in situ* using a digital camera–type Canon EOS 60D. Macromorphological characteristics were recorded on fresh materials, according to Tulloss and Yang (2011). Color codes recorded from fresh materials follow Kornerup and Wanscher (1981). The fresh basidiomata were dried using an electric dryer Stockli Dorrex at 45°C for 1 day and stored thereafter as exsiccates with their label in sealable plastic bag–type minigrip. The dried specimens along with the holotype of the newly described species are deposited in the Mycological Herbarium of the University of Parakou (UNIPAR). Duplicates of dried specimens and the isotype of the new species are conserved at the Herbarium of Cryptogams of Kunming Institute of Botany, Chinese Academy of Sciences (KUN-HKAS). Small pieces of fresh basidiomata were also stored in CTAB lysis buffer (2% cetyl trimethylammonium bromide, 100 mM Tris–HCl, 20 mM EDTA, 1.4 M NaCl) and dried with silica gel for molecular investigations. Nomenclature aspects, as well as authorities for scientific names, have been double checked against Index Fungorum^1^ and in Tulloss et al. (2020).

### Micromorphological Investigations

Microscopic structures were studied from dried materials mounted in 5% KOH and stained with Congo red to depict all tissues. The Melzer’s reagent was used to test the amyloidity of basidiospores. All measurements and line drawings were performed at 1,000× magnification, and a minimum of 20–30 basidiospores from each basidioma were measured in side view. Micromorphological investigations were performed by mean of a microscope-type Nikon Eclipse 50i. The abbreviation (n/m/p) is used to describe basidiospores where n is the number of basidiospores from m basidiomata of p collections. The basidiospores dimensions are provided using the notation (a)b – c(d) with the range b–c containing a minimum of 95% of the measured values, a and d in the brackets showing the two extreme values. Q is used for the ratio length/width of a spore in side view; Qm is the average Q of all basidiospores ± sample standard deviation. The measurements for the basidiospores were analyzed with Piximetre v5.10 (Henriot and Cheype, 2020). The descriptive terms are in accordance with Bas (1969), Yang (2005), Yang (2015), Cai et al. (2016), and Cui et al. (2018).

### DNA Extraction, Amplification, and Sequencing

The total genomic DNA was obtained from materials preserved in CTAB or dried with silica gel following the modified CTAB procedure (Doyle and Doyle, 1987). Polymerase chain reaction (PCR) amplification and sequencing were performed in accordance with those described in Cai et al. (2014) and Cui et al. (2018). The following primer pairs were used for PCR amplification and sequencing: ITS1F and ITS4 to amplify ITS region (White et al., 1990; Gardes and Bruns, 1993); LROR and LR5 (Vilgalys and Hester, 1990) for nrLSU region; EF1-983F and EF1-1567R (Rehner and Buckley, 2005) for the translation elongation factor 1-α (*tef1-*α) region; ARPB2-6F and ARPB2-7R for RNA polymerase II second largest subunit (*rpb2*) region; and Am-b-tubulin F and Am-b-tubulin R (Cai et al., 2014) for beta-tubulin (β*-tubulin*) region.

### Sequence Alignment and Phylogenetic Analyses

Thirty-four sequences (seven for ITS, eight for nrLSU, seven for *rpb2*, six for *tef1-*α, and six for β*-tubulin*) were newly generated for this study and deposited in GenBank (Table 1)^2^. Additional sequences were retrieved from previously published articles and GenBank (Table 1). The sequences were aligned using MAFFT v7.310 (Katoh and Standley, 2013) and edited manually when necessary using BioEdit v7.0.9 (Hall, 1999). The poorly aligned portions and divergent regions were eliminated using Gblocks v0.91b (Castresana, 2000; Talavera and Castresana, 2007). A concatenated dataset (including ITS, nrLSU, *rpb2*, *tef1-*α, β*-tubulin*) comprising 312 sequences was constructed using Phyutility v2.2 (Smith and Dunn, 2008) and used for phylogenetic analyses. Before using the concatenated dataset for phylogenetic analyses, the Incongruence Length Difference test in PAUP v4.0a168 (Swofford, 2002) was performed to detect any conflicts between the gene regions. As no incongruence (*P* = 0.363000) was detected, the maximum likelihood (ML) and Bayesian inference (BI) were used on the concatenated alignment for phylogenetic tree inference. The ML analysis was performed using RAxML v7.9.1 (Stamatakis, 2006) under the GTR + GAMMA + I nucleotide substitution model and performing non-parametric bootstrapping with 1,000 replicates. The BI was performed in MrBayes v3. 2 (Ronquist et al., 2012). The best substitution model was determined using the Akaike Information Criterion implemented in jModeltest v.2 on the CIPRES Science Gateway v3.1 (Miller et al., 2010). The BI was conducted with the following parameters: two runs, each with four simultaneous Markov chains, and trees were summarized every 1,000 generations. The analyses were completed after 20,000,000 generations when the average standard deviation of split frequencies was 0.002200 for the five-gene analysis, and the first 25% generations were discarded as burn-in. The phylograms from ML and BI analyses were visualized with FigTree v1.4.3 (Rambaut, 2009) and then edited in Adobe Illustrator CS6.

**TABLE 1:** Taxa of *Amanita* included in molecular phylogenetic analyses.

Species name	Collection or collector no.	Country of origin	GenBank accession no.				
			ITS	nrLSU	*rpb2*	*tef1-*α	β*-tubulin*
*Amanita alliodora*	DSN062	Madagascar	KX185611	KX185612	–	–	–
***Amanita albolimbata***	JEIC0707	Benin	MT966936	MT966943	MT966959	MT966956	MT966951
***Amanita albolimbata***	JEIC0739	Benin	MT966935	MT966942	MT966963	MT966955	MT966950
***Amanita albolimbata***	JEIC0667	Benin	MT966932	MT966939	MT966958	MT966953	MT966947
***Amanita albolimbata***	JEIC0675	Benin	MT966934	MT966941	MT966962	–	MT966949
***Amanita albolimbata***	JEIC0653	Benin	MT966933	MT966940	MT966961	MT966954	MT966948
***Amanita albolimbata***	JEIC0638	Benin	MT966931	MT966938	MT966960	MT966952	MT966946
***Amanita albolimbata***	HKAS94241	Benin	–	MT966944	MT966964	MT966957	–
***Amanita albolimbata***	HKAS93847	Benin	MT966937	MT966945	–	–	–
*Amanita bisporigera*	RET377-9	United States	KJ466374	KJ466434	–	KJ481936	KJ466501
*Amanita brunneitoxicaria*	BZ2015-01	Thailand	KY747462	–	KY656879	–	KY656860
*Amanita brunneitoxicaria*	BZ2015-02	Thailand	KY747463	–	KY656880	–	KY656861
*Amanita bweyeyensis*	JD 1304	Rwanda	MK570920	MK570927	MK570937	MK570940	MK570916
*Amanita bweyeyensis*	JD 1257	Rwanda	MK570919	MK570926	–	–	–
*Amanita bweyeyensis*	TS 591	Tanzania	MK570921	MK570928	–	–	–
*Amanita djarilmari*	PERTH08776067	Australia	KY977732	KY977704	MF000755	MF000750	MF000742
*Amanita djarilmari*	PERTH08776059	Australia	–	KY977705	MF000754	–	–
*Amanita djarilmari*	PERTH08776040	Australia	–	KY977708	–	MF037234	MF000743
*Amanita eucalypti*	PERTH8809828	Australia	–	KY977707	MF000758	MF000751	MF000746
*Amanita eucalypti*	PERTH8809860	Australia	–		MF000757	–	MF000745
*Amanita eucalypti*	PERTH8809763	Australia	–	KY977709	–	–	MF000747
*Amanita exitialis*	HKAS74673	China	KJ466375	KJ466435	KJ466590	KJ481937	KJ466502
*Amanita exitialis*	HKAS75774	China	JX998027	JX998052	KJ466591	JX998001	KJ466503
*Amanita exitialis*	HKAS75775	China	JX998026	JX998053	KJ466592	JX998002	KJ466504
*Amanita exitialis*	HKAS75776	China	JX998025	JX998051	KJ466593	JX998003	KJ466505
*Amanita fuliginea*	HKAS75780	China	JX998023	JX998048	KJ466595	JX997995	KJ466507
*Amanita fuliginea*	HKAS75781	China	JX998021	JX998050	KJ466596	JX997994	KJ466508
*Amanita fuliginea*	HKAS75782	China	JX998022	JX998049	KJ466597	JX997996	KJ466509
*Amanita fuliginea*	HKAS79685	China	KJ466377	KJ466437	KJ466594	KJ481938	KJ466506
*Amanita fuligineoides*	HKAS83694	China	–	MH486553	MH486020	MH508824	MH485540
*Amanita fuligineoides*	HKAS52727	China	JX998024	JX998047	KJ466599	–	KJ466511
*Amanita gardneri*	PERTH08776121	Australia	KU057387	KY977712	MF000756	MF000752	MF000748
*Amanita griseorosea*	HKAS89004	China	–	KU168387	KU168388	KU168386	KU168389
*Amanita griseorosea*	HKAS77332	China	KJ466411	KJ466474	–	KJ481992	KJ466578
*Amanita griseorosea*	HKAS77333	China	KJ466412	KJ466475	KJ466660	KJ481993	KJ466579
*Amanita harkoneniana*	P Pirot SN	Madagascar	MK570922	MK570929	MK570938	MK570941	MK570917
*Amanita harkoneniana*	TS 1061	Tanzania	MK570923	MK570930	–	–	–
*Amanita marmorata*	HW SN	Australia	MK570924	MK570931	MK570939	MK570942	MK570918
*Amanita marmorata*	RET 623-7	Australia	KP757875	KP757874	–	–	–
*Amanita millsii*	HO581533	Australia	KY977714	KY977713	MF000753	MF000759	MF000760
*Amanita molliuscula*	HKAS77324	China	KJ466409	KJ466472	KJ466639	KJ481974	KJ466553
*Amanita molliuscula*	HKAS75555	China	KJ466408	KJ466471	KJ466638	KJ481973	KJ466552
*Amanita molliuscula*	HMJAU20469	China	KJ466410	KJ466473	KJ466640	KJ481975	KJ466554
*Amanita ocreata*	HKAS79686	United States	KJ466381	MH486688	KJ466607	KJ481947	KJ466518
*Amanita pallidorosea*	HKAS82350	China	MH508485	MH486737	MH486163	MH508971	MH485668
*Amanita pallidorosea*	HKAS75483	China	KJ466384	KJ466445	KJ466623	KJ481959	KJ466535
*Amanita pallidorosea*	HKAS75786	China	JX998037	JX998054	KJ466627	JX998011	KJ466539
*Amanita pallidorosea*	HKAS77349	China	KJ466389	KJ466449	KJ466628	KJ481961	KJ466540
*Amanita parviexitialis*	HKAS79049	China	–	KT971342	KT971345	KT971343	KT971346
*Amanita parviexitialis*	HKAS79601	China	–	–	–	KT971344	KT971347
*Amanita phalloides*	MB-102659	Germany	–	MH486754	–	–	–
*Amanita phalloides*	HKAS75773	United States	JX998031	JX998060	KJ466612	JX998000	KJ466523
*Amanita phalloides*	Qs6	France	–	–		EU886739	–
*Amanita reidii*	AY325883	South Africa	–	AY325883	–	–	–
*Amanita rimosa*	HKAS101393	China	–	MH486806	MH486218	MH509031	MH485722
*Amanita rimosa*	HKAS77335	China	KJ466393	KJ466455	KJ466621	KJ481957	KJ466532
*Amanita rimosa*	HKAS77279	China	KJ466392	KJ466454	KJ466620	KJ481956	KJ466531
*Amanita rimosa*	HKAS77120	China	MH508547	KJ466453	KJ466619	KJ481955	KJ466530
*Amanita suballiacea*	RET491-7	United States	KJ466421	KJ466486	KJ466602	KJ481942	KJ466514
*Amanita suballiacea*	RET478-6	United States	KJ466419	KJ466484	KJ466600	KJ481940	KJ466512
*Amanita suballiacea*	RET490-1	United States	KJ466420	KJ466485	KJ466601	KJ481941	KJ466513
*Amanita subjunquillea*	HKAS75770	China	JX998034	JX998062	KJ466653	JX997999	KJ466571
*Amanita subjunquillea*	HKAS75771	China	JX998032	JX998063	KJ466654	JX997997	KJ466572
*Amanita subjunquillea*	HKAS75772	China	JX998033	JX998061	KJ466655	JX997998	KJ466573
*Amanita subjunquillea*	HKAS77325	China	KJ466425	KJ466490	KJ466656	KJ481988	KJ466574
*Amanita subpallidorosea*	LHJ140923-41	China	KP691683	KP691692	KP691701	KP691670	KP691711
*Amanita subpallidorosea*	LHJ140923-55	China	KP691680	KP691693	KP691702	KP691671	KP691712
*Amanita subpallidorosea*	LHJ140926-11	China	KP691682	KP691688	KP691703	KP691672	KP691708
*Amanita virosa*	HKAS90176	China	MH508650	MH486948	MH486341	MH509167	MH485847
*Amanita virosa*	HKAS56694	Finland	JX998030	JX998058	KJ466664	JX998007	KJ466583
*Amanita virosa*	HMJAU23303	China	KJ466430	KJ466497	KJ466666	KJ481998	KJ466586
*Amanita virosa*	HKAS71040	Japan	KJ466429	KJ466496	KJ466665	KJ481997	KJ466584
**Outgroup**							
*Amanita zangii*	HKAS99663	China	MH508655	MH486958	MH486351	MH509178	MH485855
*Amanita zangii*	GDGM29241	China	KJ466432	KJ466499	KJ466668	KJ482000	KJ466588
*Amanita hesleri*	RET 155-1	United States	–	HQ539701	–	–	–

*The new sequences generated in this study are highlighted in bold font. References to sequences retrieved from GenBank: Cai et al. (2014, 2016), Thongbai et al. (2017); Cui et al. (2018), and Fraiture et al. (2019).*

### Analysis of Toxins by Liquid Chromatography–High-Resolution Mass Spectrometry

Dried basidiomata of the target taxon have been used for toxin analyses using the method of Lüli et al. (2019). Toxins were extracted from basidiomata, using methanol–water–0.01 M hydrochloric acid (5:4:1, vol/vol) as the extraction buffer. Dried material (0.05 g) was crushed into fine powder in a mortar and pestle with liquid nitrogen. Then, 1.5 mL aforementioned buffer was added, and the suspension transferred into 1.5-mL centrifuge tubes. The tubes were kept at room temperature for 30 min, followed by centrifugation (12,000 rpm) for 3 min. Finally, the supernatant was transferred into new centrifuge tubes for mass spectrometry analysis.

The presence of cyclic peptides, especially α-amanitin, β-amanitin, phalloidin, and phallacidin (standards provided by Sigma Chemical Co, United States), was evaluated through the liquid chromatography–high-resolution mass spectrometry (LC-HRMS) using 1290 Infinity II HPLC systems coupled with 6540 UHD precision mass Q-TOF instruments under the conditions listed in Table 2.

**TABLE 2:** Instrument parameters for the UHPLC-MS analyses.

Parameter	Value
HPLC parameters	
Analytical column	C18, 4.6 × 100 mm I.D., particle size 2.7 μm, Agilent Technologies
Column temperature	28°C
Mobile phase A	0.02 M aqueous ammonium acetate-acetonitrile (90:10, vol/vol)
Mobile phase B	100% acetonitrile
Injection volume	10 μL
Binary pump gradient	Time (min) %A %B Flow (mL/min)
and flow	0.00 100 0 0.500
	2.00 100 0 0.500
	10.00 0 100 0.500
Electrospray MS parameters	
Ionization mode	Positive ESI
Scan range	500–1,700
Gas temperature	350°C
Gas flow	8 L/min (N_2_)
Capillary voltage	3.5 kV

## Results

### Phylogenetic Data

**FIGURE 2:**
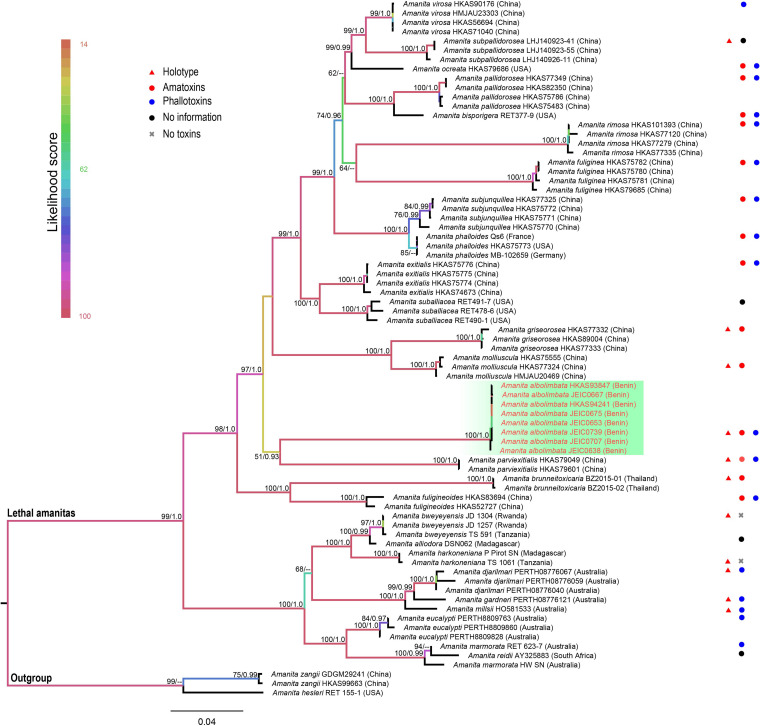
Phylogenetic relationship and placement of *Amanita albolimbata* within *A.* sect. *Phalloideae* inferred from the combined dataset (ITS, nrLSU, *rpb2*, *tef1-*α, and β*-tubulin)* using maximum likelihood (ML). Bootstrap values ≥50% and Bayesian posterior probabilities ≥0.90 are reported on branches. Sequences generated in this study are highlighted in red.

The topologies of ML and BI phylogenetic trees obtained in this study are practically the same (Supplementary Figures S1, S2). In the combined dataset (ITS, nrLSU, *rpb2*, *tef1-*α, and β*-tubulin*), 312 sequences were included. The combined dataset contained 3,024 total characters, including 2,070 constant (proportion = 0.684524), 93 variable and parsimony-uninformative, and 861 parsimony-informative. The target species, *A. albolimbata*, forms a well-supported distinct lineage (MLB = 100%, BPP = 1.0) and is a close sister to the Asian species (*A. parviexitialis* Qing Cai, Zhu L. Yang and Yang-Yang Cui) (Figure 2 and see also Supplementary Figures S1–S7). Within the section, *A. albolimbata* is genetically distant from other African species such as *A. alliodora*, *A. bweyeyensis*, and *A. harkoneniana*. In the phylogenetic tree, the African and Australian species hold the basal positions.

### Taxonomy

*Amanita albolimbata* J.E.I. Codjia, N.S. Yorou and Zhu L. Yang, sp. nov.

Mycobank: MB836777

Figures 3, 4

**FIGURE 3:**
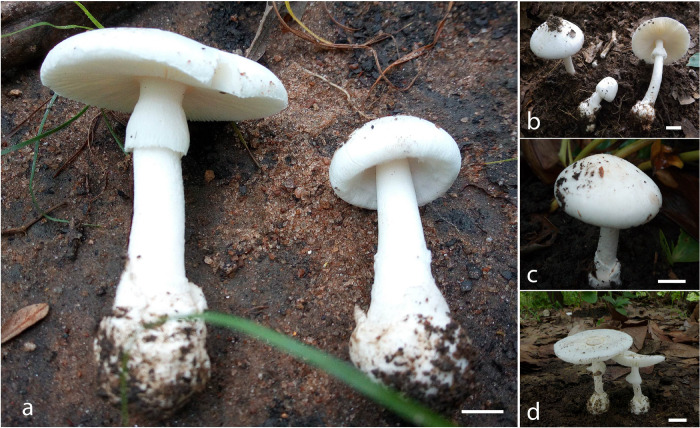
Basidiomata of *A. albolimbata* [**(a)** holotype JEIC0739; **(b)** JEIC0653; **(c)** JEIC0667; **(d)** HKAS93847]. Scale bar: 1 cm (photos by Codjia J.E.I. and Gang Wu).

**FIGURE 4:**
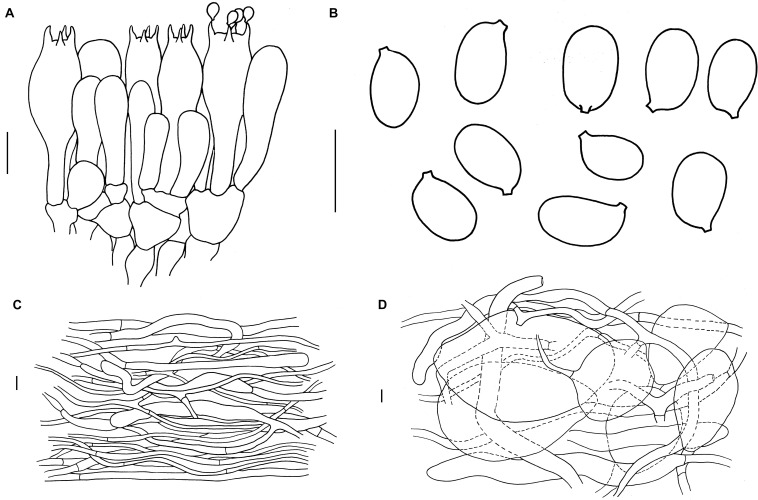
Microscopic features of *A. albolimbata*. Holotype JEIC0739 [**(A)** hymenium and subhymenium; **(B)** basidiospores]. HKAS93847 [**(C)** pileipellis; **(D)** elements of inner part of volva]. Scale bars: a–d = 10 μm.

#### Etymology

“Albo” (white), “limbata” (limb bearing volva), meaning white limbate volva.

#### Type

BENIN. Donga Province: Bassila, 09°07′58″N, 2°07′43″E, Forest Reserve of Bassila, woodland of *Uapaca togoensis* Pax (Phyllanthaceae), date: 06 August 2019, leg. and det. Jean Evans I. CODJIA, Holotype JEIC0739 (UNIPAR), Isotype (KUN-HKAS 107736), GenBank Acc. No.: (ITS = MT966935, nrLSU = MT966942, rpb2 = MT966963, tef1-α = MT966955, β-tubulin = MT966950).

#### Basidiomata Small-Sized

Pileus 38–58 mm in diameter, hemispherical when young and then expanding to regularly convex or applanate at the maturity, smooth, without umbo, subviscid when moist, sometimes the presence of patchy volval remnants, white (1A1); margin non-striate, non-appendiculate, white (1A1). Lamellae free, white (1A1), lamellulae attenuate. Stipe 55–87 mm long × 6–13 mm diameter, white, nearly cylindrical, covered with white (1A1) squamules. Annulus present, superior, white (1A1), membranous. Basal bulb of the stipe globose, surrounded by a white (1A1) limbate membranous volva (also white inside). Context stuffed, white (1A1). Odor and taste not recorded.

#### Lamellar Trama Bilateral

Mediostratum 36–50 μm wide, composed of abundant subfusiform to ellipsoidal inflated cells (30–90 × 15–30 μm), and abundant filamentous hyphae, 3–4 μm wide; vascular hyphae scarce. Lateral stratum composed of ellipsoid to subglobose inflated cells (25–40 × 20–30 μm), diverging at an angle of ca. 30°–45° to mediostratum; filamentous hyphae abundant, 3–9 μm wide. Subhymenium 35–45 μm thick, with 2–3 layers of ovoid to subglobose or irregular cells, 9–12 × 8–10 μm. Basidia (Figure 3a) [40/4/4] 30–48 × 9.5–11 (–14) μm, clavate, 4-spored; basal septa lacking clamps. Basidiospores (Figure 3b) [90/4/4] (7.5–) 9 (–11) × (5–) 6–7 (–7.5) μm, *Q* = (1.2) 1.4–1.5 (1.7), *Qm* = 1.4 ± 0.05, broadly ellipsoid to ellipsoid, smooth, colorless, amyloid. Lamellar edge sterile, composed of subglobose to ellipsoid, inflated cells (15–40 × 10–25 μm), filamentous hyphae, 3–9 μm wide, irregularly arranged or running parallel to lamellar edge. Pileipellis 250–370 μm thick, 2-layered; suprapellis up to 100–170 μm thick, slightly gelatinized, composed of arranged, thin-walled, colorless to nearly colorless, ellipsoid to clavate terminal cells 80–180 × 10–20 μm, mixed with filamentous hyphae 3–5 μm wide; subpellis up to 150–200 μm thick, composed of undifferentiated, filamentous hyphae 2–5 μm wide; vascular hyphae scarce. Inner part of volva composed of longitudinally arranged elements: filamentous hyphae abundant 9- to 15-μm-wide, colorless, thin to slightly thick-walled branching; inflated cells abundant, subglobose to ellipsoid, 60–155 × 35–90 μm, colorless, thin to slightly thick-walled, terminal, mixed with long clavate terminal cells 130–210 × 15–20 μm; vascular hyphae rare. Outer part of volva similar to the inner part of volva but with less abundant inflated cells. Stipe trama composed of longitudinally arranged, long clavate terminal cells 130–540 × 15–25 μm; filamentous hyphae scattered to abundant, 3–10 μm wide; vascular hyphae scarce. Clamps absent in all tissues.

#### Habitat

Solitary, rarely or in small group of 2 or 3 individuals, on the ground in woodland and gallery forests, associated with *Uapaca guineensis* Müll. Arg. or *U. togoensis* (Phyllanthaceae) and *Isoberlinia doka* Craib and Stapf (Fabaceae/Leguminosae).

#### Distribution

Currently known from Benin, but likely occurs more widely in the region in similar vegetation.

Additional specimens examined were BENIN. Colline Province: Ouèssè, 08°26′34.4.0″ N, 02°33′09.0″ E, open forest, date: 02 July 2015, leg. B. Feng 1854 (HKAS94241), date: 03 July 2015, leg. G. Wu 1470 (HKAS93847). Borgou Province: Ndali, 09°14′31.93″N, 02°43′22.7″E, Forest Reserve of Ndali, date: 31 July 2019, leg. and det. Jean Evans I. CODJIA, JEIC0638. Donga Province: Bassila, 09°07′58″N, 2°07′43″E, Forest Reserve of Bassila, date: 02 August 2019, leg and det. Jean Evans I. CODJIA, JEIC0707. Atacora Province: Kota, 10°12′39″ N, 01°26′45.8″ E, gallery forest of Kota, date: 08 August 2019, leg. and det. Jean Evans I. CODJIA, JEIC0653. Borgou Province: Okpara, 09°16′34.8″N, 02°43’12.8″E, Forest Reserve of Okpara, date: 22 August 2019, leg. and det. Jean Evans I. CODJIA, JEIC0667, date: 11 September 2019, leg. and det. Jean Evans I. CODJIA, JEIC0675.

### Analysis of Toxins by LC-HRMS

**FIGURE 5:**
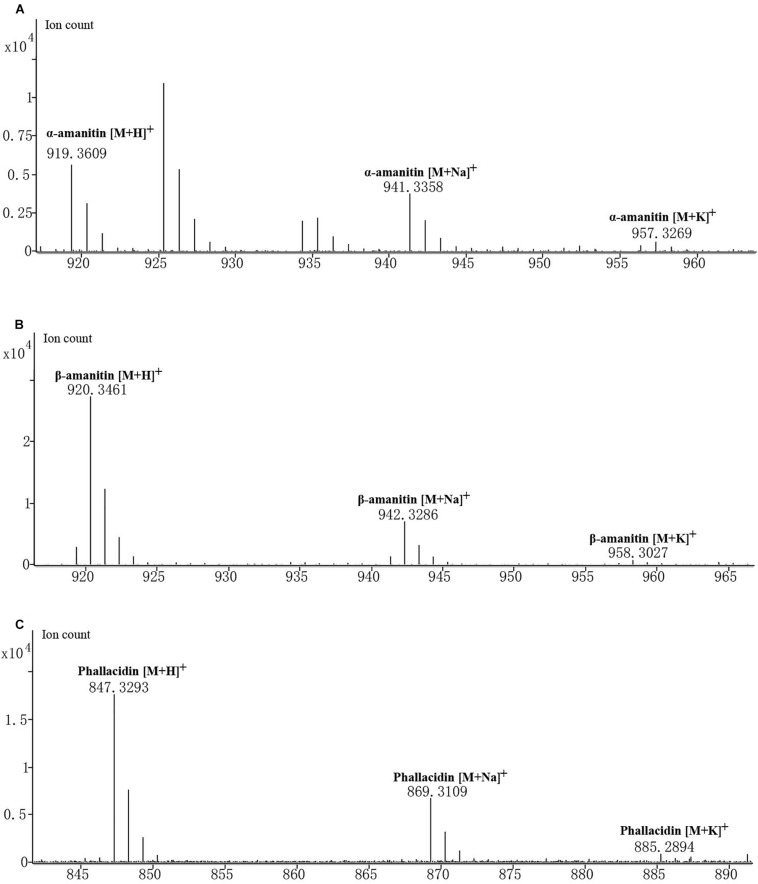
Mass spectrogram of cyclic peptides produced in *A. albolimbata*. **(A)** α-Amanitin. **(B)** β-Amanitin. **(C)** Phallacidin.

*Amanita albolimbata* contains three cyclic peptides: α-amanitin, β-amanitin, and phallacidin (Figure 5). The formula of α-amanitin is C_3__9_H_5__4_N_1__0_O_1__4_S with a monoisotopic mass of 918.3541. The calculated mass of the [M + H]^+^ ion is 919.3614, and the measured mass was 919.3609 with mass discrepancy of 0.59 ppm. The formula of β-amanitin is C_3__9_H_5__3_N_9_O_1__5_S with a monoisotopic mass of 919.3381. The calculated mass of the [M + H]^+^ ion is 920.3455, and the measured mass was 920.3461 with mass discrepancy of 0.7 ppm. The formula of phallacidin is C_3__7_H_5__0_N_8_O_1__3_S with a monoisotopic mass of 846.3218. The calculated mass of the [M + H]^+^ ion is 847.3291, and the measured mass was 847.3293 with mass discrepancy of 0.26 ppm. The measured masses of the two other adduct ions for above cyclic peptides, [M + Na]^+^ and [M + K]^+^, are also included in Figure 5. No corresponding mass was identified for phalloidin.

## Discussion

### Species Delimitation

Most of the fatal mushroom poisonings are caused by lethal amanitas belonging to *A.* sect. *Phalloideae* (Bresinsky and Besl, 1990; Unluoglu and Tayfur, 2003; Cai et al., 2014, 2016; Li et al., 2015; Cui et al., 2018). In tropical Africa, very few lethal amanitas have been reported (Walleyn and Verbeken, 1998; Fraiture et al., 2019; Tulloss et al., 2020). Only six species are known from tropical Africa including *A. alliodora*, *A. murinacea*, and *A. thejoleuca* described from Madagascar and *A. bweyeyensis, A. harkoneniana*, and *A. strophiolata* described from DR Congo (central Africa). *Amanita albolimbata* represents a new lethal *Amanita* from tropical Africa and can be recognized by its white basidiomata with a convex or applanate pileus without umbo, a limbate volva with inner part composed of abundant inflated cells, and broadly ellipsoid to ellipsoid basidiospores.

The multigene phylogenetic analyses revealed that *A. albolimbata* is an independent lineage in *A. sect. Phalloideae*. Surprisingly, the species is genetically distant from other African species such as *A. alliodora*, *A. bweyeyensis*, and *A. harkoneniana* that form a clade. Among lethal amanitas from tropical Africa, *A. albolimbata* is similar to *A. strophiolata* because of the white basidiomata, but unfortunately, we do not have any material of the latter species to test its phylogenetic relationship to other species. However, *A. strophiolata* presents some distinct morphological characteristics that clearly separate it from *A. albolimbata*. *Amanita strophiolata* was described by Beeli (1927, 1935) from DR Congo and is distinguished by its umbonate pileus, often yellowish at center, the absence of volval remnants on pileus, ellipsoid to elongate basidiospores (10–11 × 6–7 μm), a distinctive annulus in the form of a funnel, and larger basidiomata.

*Amanita albolimbata* also shows some similarities with Asian, European, and American species including *A. exitialis, A. bisporigera, A. molliuscula*, *A. parviexitialis*, and *A. virosa*, based on the white basidiomata. Those species have never been reported in tropical Africa and differ from the African species by morphological characteristics. *Amanita exitialis* was described from China (Yang and Li, 2001) and subsequently has been collected from India (Bhatt et al., 2003). The species is distinguished from *A. albolimbata* by the absence of voval remnants on pileus, globose-to-subglobose basidiospores (9.5–12 × 9–11.5 μm), 2-spored basidia, scarce inflated cells in the inner part of the volva, and larger basidiomata (Yang and Li, 2001; Cui et al., 2018). *Amanita bisporigera* was described from North America and characterized by the absence of voval remnants on pileus, a skirt-like annulus, globose-to-subglobose basidiospores (7.8–9.6 × 7–9 μm), 2-spored basidia and sometimes 4-spored, and larger basidiomata (Jenkins, 1986; Tulloss et al., 1995; Yang and Li, 2001; Yang, 2015). The species mainly has 2-spored basidia, in late spring and early summer; later in year, it may sometimes mainly or entirely have 4-spored basidia (Tulloss and Possiel, 2005). *Amanita molliuscula* was described from China and characterized by the absence of voval remnants on pileus, globose-to-subglobose basidiospores (7.5–9 × 7–8 μm), and larger basidiomata (Cai et al., 2016; Cui et al., 2018). *Amanita parviexitialis* was described from China and characterized by the absence of voval remnants on pileus sometimes slightly brownish at center, subglobose, rarely globose to broadly ellipsoidal basidiospores (7.5–9.5 × 7–9 μm), 2-spored basidia, and smaller basidiomata (Cai et al., 2016; Cui et al., 2018). *Amanita virosa* is widely distributed across Europe and temperate to subtropical Asia (Neville and Poumarat, 2004; Zhang et al., 2010; Li et al., 2015; Yang, 2015; Cai et al., 2016; Cui et al., 2018). It has an umbonate pileus, white, often cream at the center, the absence of volval remnants on pileus, the presence of globose-to-subglobose basidiospores (8–11 × 8–10 μm), scarce inflated cells in the inner part of the volva, and larger basidiomata (Cai et al., 2016; Cui et al., 2018). Generally, *A. albolimbata* sometimes has a patchy volval remnant on the pileus, which is typically absent for *A. exitialis*, *A. bisporigera*, *A. molliuscula*, *A. strophiolata*, and *A. virosa* (Beeli, 1927, 1935; Tulloss and Possiel, 2005; Cai et al., 2016; Cui et al., 2018).

*Amanita albolimbata* is also distinct from other white species in *Amanita* sect. *Phalloideae* by its ecology. It occurs in woodland and gallery forests, associated with *U. guineensis* or *U. togoensis* (Phyllanthaceae) and *I. doka* (Fabaceae/Leguminosae), whereas *A. strophiolota* grows in swampy forests (Beeli, 1927, 1935). *Amanita exitialis*, *A. bisporigera*, *A. molliuscula*, *A. parviexitialis*, and *A. virosa* occur in forests of Pinaceae and Fagaceae (Tulloss et al., 1995; Cai et al., 2016; Cui et al., 2018).

In the multigene phylogenetic tree, the African and Australian taxa are basal. This suggests that the lethal amanitas originated from the palaeotropical areas. Cai et al. (2014) also suggested a possible palaeotropical origin of lethal amanitas and highlighted the need for more molecular–phylogenetic studies on collections from the tropics and the Southern Hemisphere.

### Toxicity in *Amanita*

For centuries, wild mushrooms have been consumed massively and popular in the human diet because of their matchless taste, protein content, and medicinal properties (de Román et al., 2006; Cheung, 2010). However, the high interest on wild mushroom collections and consumption could increase the risk of poisoning by lethal mushrooms. During picking, confusions could easily be made between edible and poisonous mushrooms because of their morphological similarities. Many mushroom poisoning cases have been reported worldwide and have mainly been caused by members of *A.* sect. *Phalloideae* (Zhang et al., 2010; Cai et al., 2014, 2016; Yang, 2015; Li et al., 2015, 2020; Thongbai et al., 2017). Consequently, much attention has been devoted to the species producing toxins within *A.* sect. *Phalloideae* (Chen et al., 2014; Li et al., 2014; Garcia et al., 2015; Cai et al., 2016).

The different toxins documented in those species are mainly amatoxins, phallotoxins, and virotoxins, which can cause severe damages, like liver and renal failure (Wieland, 1973, 1986; Chen et al., 2014). *Amanita exitialis*, *A. bisporigera*, *A. brunneitoxicaria*, *A. djarilmari*, *A. eucalypti*, *A. fuliginea*, *A. fuligineoides*, *A. gardeneri*, *A. marmorata*, *A. millsii*, *A. molliuscula*, *A. ocreata*, *A. pallidorosea*, *A. parviexitialis*, *A. phalloides*, *A. rimosa*, *A. suballiacea*, *A. subjunquillea*, *A. verna*, and *A. virosa* are known to contain those toxins and are distributed across Asia, America, Australia, and Europe (Wieland, 1973, 1986; Bresinsky and Besl, 1985; Chen et al., 2014; Li et al., 2014; Garcia et al., 2015; Cai et al., 2016).

Until now, no lethal amanitas had been reported from West Africa. However, lethal amanitas have been documented from Central Africa and Madagascar (Fraiture et al., 2019). *Amanita albolimbata* represents the first lethal species of *A.* sect. *Phalloideae* known from West Africa. The most notorious toxins, α-amanitin, β-amanitin, and phallacidin, have also been detected in the species.

Numerous amanitoid taxa are harvested and consumed by local people in tropical Africa (Codjia and Yorou, 2014; Yorou et al., 2014; Boni and Yorou, 2015; De Kesel et al., 2017; Fadeyi et al., 2017; Milenge et al., 2018; Soro et al., 2019). Because of the whitish color of the basidiomata, *A. albolimbata* can be confused with *A. subviscosa* Beeli. *Amanita subviscosa* is commonly harvested and used as food by local people in Benin (Yorou et al., 2014; Boni and Yorou, 2015; Fadeyi et al., 2017, 2019). Still, *A. subviscosa* displays contrasting morphological characteristics with *A. albolimbata* by a slightly squamulose and viscous pileus, slightly striated margin, slightly bulbous, and slightly furfuraceous and hollow stipe, with a distinctive membranous volva (Beeli, 1935). The lack of *A. albolimbata* in various ethnomycological investigations (Yorou et al., 2014; Boni and Yorou, 2015; Fadeyi et al., 2017; Soro et al., 2019) attests that either local people are aware about its toxicity, or some fatal but unrecorded cases did occur within rural communities. However, it is important to educate local people on the best ways to discriminate morphologically close taxa in order to avoid the consumption of lethal *Amanita* species for an effective prevention of future poisoning incidents.

## Data Availability Statement

The datasets generated for this study can be found in the online repositories. The names of the repository/repositories and accession number(s) can be found in the article/Supplementary Material.

## Author Contributions

ZLY, JEIC, and NSY conceived and designed the research. JEIC collected the species, performed the molecular phylogenetic analyses and the taxonomic studies, and wrote the first draft of the manuscript. JEIC and QC generated the DNA sequences. JEIC and SWZ carried out the cyclic peptide toxins analyses. QC, HL, MR, NSY, and ZLY critically revised and approved the final manuscript. All authors contributed to the article and approved the submitted version.

## Conflict of Interest

The authors declare that the research was conducted in the absence of any commercial or financial relationships that could be construed as a potential conflict of interest.

## Footnotes

1 http://www.mycology.net

2 http://www.ncbi.nlm.nih.gov/

## Supplementary Material

Supplementary Figure 1 Phylogenetic tree inferred by Maximum Likelihood analysis based on combined dataset (ITS, nrLSU, *rpb2*, *tef1-*α, and β*-tubulin*). Bootstrap values ≥50% are reported on branches. Sequences generated in this study are highlighted in red.

Supplementary Figure 2 Phylogenetic tree inferred by Bayesian Inference analysis based on combined dataset (ITS, nrLSU, *rpb2*, *tef1-*α, and β*-tubulin*). Bayesian posterior probabilities ≥0.90 are reported on branches. Sequences generated in this study are highlighted in red.

Supplementary Figure 3 Phylogenetic tree inferred by Maximum Likelihood analysis based on ITS sequences. Bootstrap values ≥50% and Bayesian posterior probabilities ≥0.90 are reported on branches. Sequences generated in this study are highlighted in red.

Supplementary Figure 4 Phylogenetic tree inferred by Maximum Likelihood analysis based on nrLSU sequences. Bootstrap values ≥50% and Bayesian posterior probabilities ≥0.90 are reported on branches. Sequences generated in this study are highlighted in red.

Supplementary Figure 5 Phylogenetic tree inferred by Maximum Likelihood analysis based on *rpb2* sequences. Bootstrap values ≥50% and Bayesian posterior probabilities ≥0.90 are reported on branches. Sequences generated in this study are highlighted in red.

Supplementary Figure 6 Phylogenetic tree inferred by Maximum Likelihood analysis based on *tef1-*α sequences. Bootstrap values ≥50% and Bayesian posterior probabilities ≥0.90 are reported on branches. Sequences generated in this study are highlighted in red.

Supplementary Figure 7 Phylogenetic tree inferred by Maximum Likelihood analysis based on β*-tubulin* sequences. Bootstrap values ≥50% and Bayesian posterior probabilities ≥0.90 are reported on branches. Sequences generated in this study are highlighted in red.
