# Supplementary figures and images for: Morphology, Multilocus Phylogeny, and Toxin Analysis Reveal Amanita albolimbata, the First Lethal Amanita Species From Benin, West Africa
Source: Front Microbiol. 2020 Nov 20;11:599047. doi: 10.3389/fmicb.2020.599047 (PMC7714729; doi:10.3389/fmicb.2020.599047)

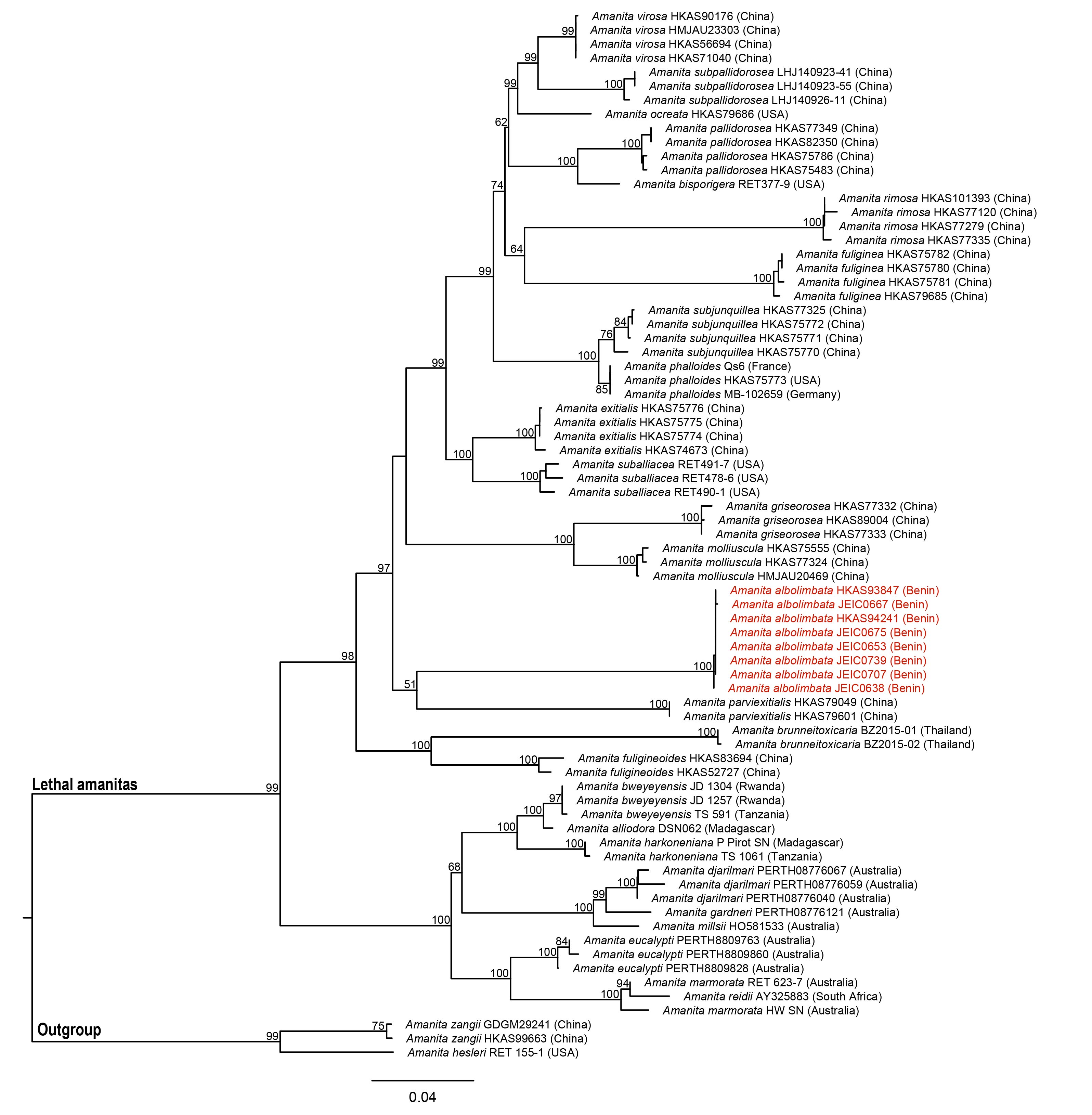

Supplement: Supplementary Figure 1 — Phylogenetic tree inferred by Maximum Likelihood analysis based on combined dataset (ITS, nrLSU, rpb2, tef1-α, and β-tubulin). Bootstrap values ≥50% are reported on branches. Sequences generated in this study are highlighted in red. [file Image_1.JPEG]

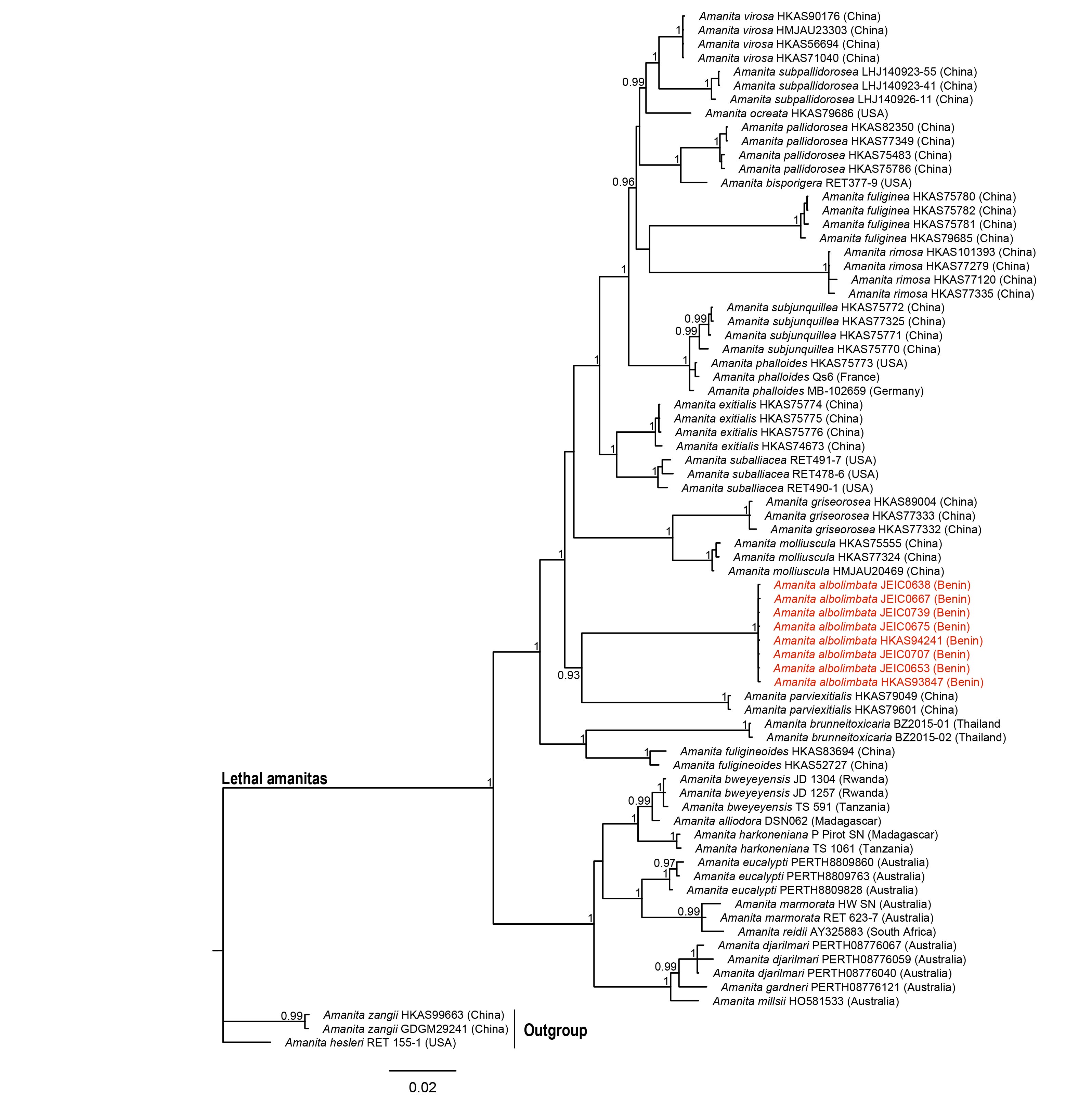

Supplement: Supplementary Figure 2 — Phylogenetic tree inferred by Bayesian Inference analysis based on combined dataset (ITS, nrLSU, rpb2, tef1-α, and β-tubulin). Bayesian posterior probabilities ≥0.90 are reported on branches. Sequences generated in this study are highlighted in red. [file Image_2.JPEG]

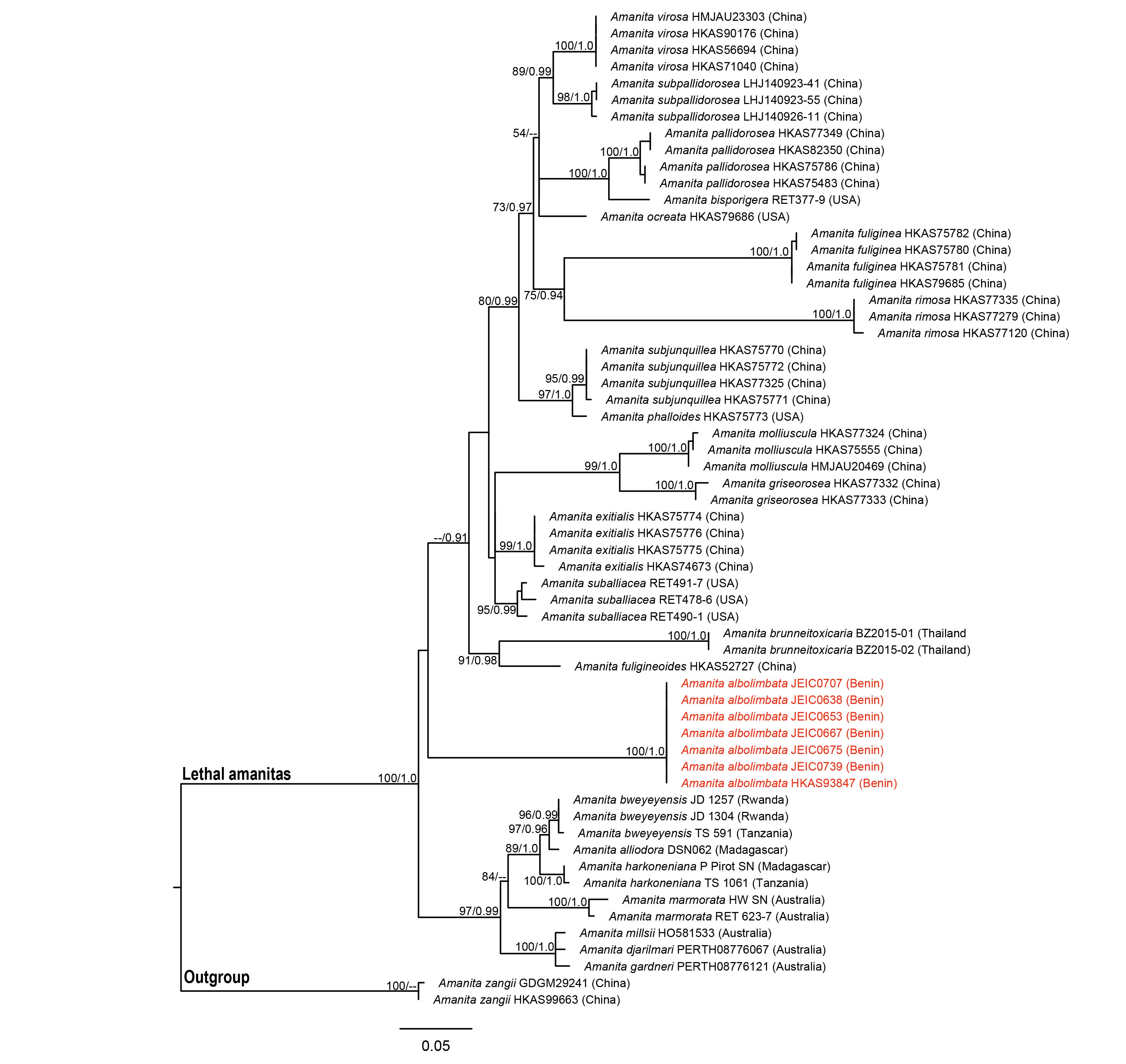

Supplement: Supplementary Figure 3 — Phylogenetic tree inferred by Maximum Likelihood analysis based on ITS sequences. Bootstrap values ≥50% and Bayesian posterior probabilities ≥0.90 are reported on branches. Sequences generated in this study are highlighted in red. [file Image_3.JPEG]

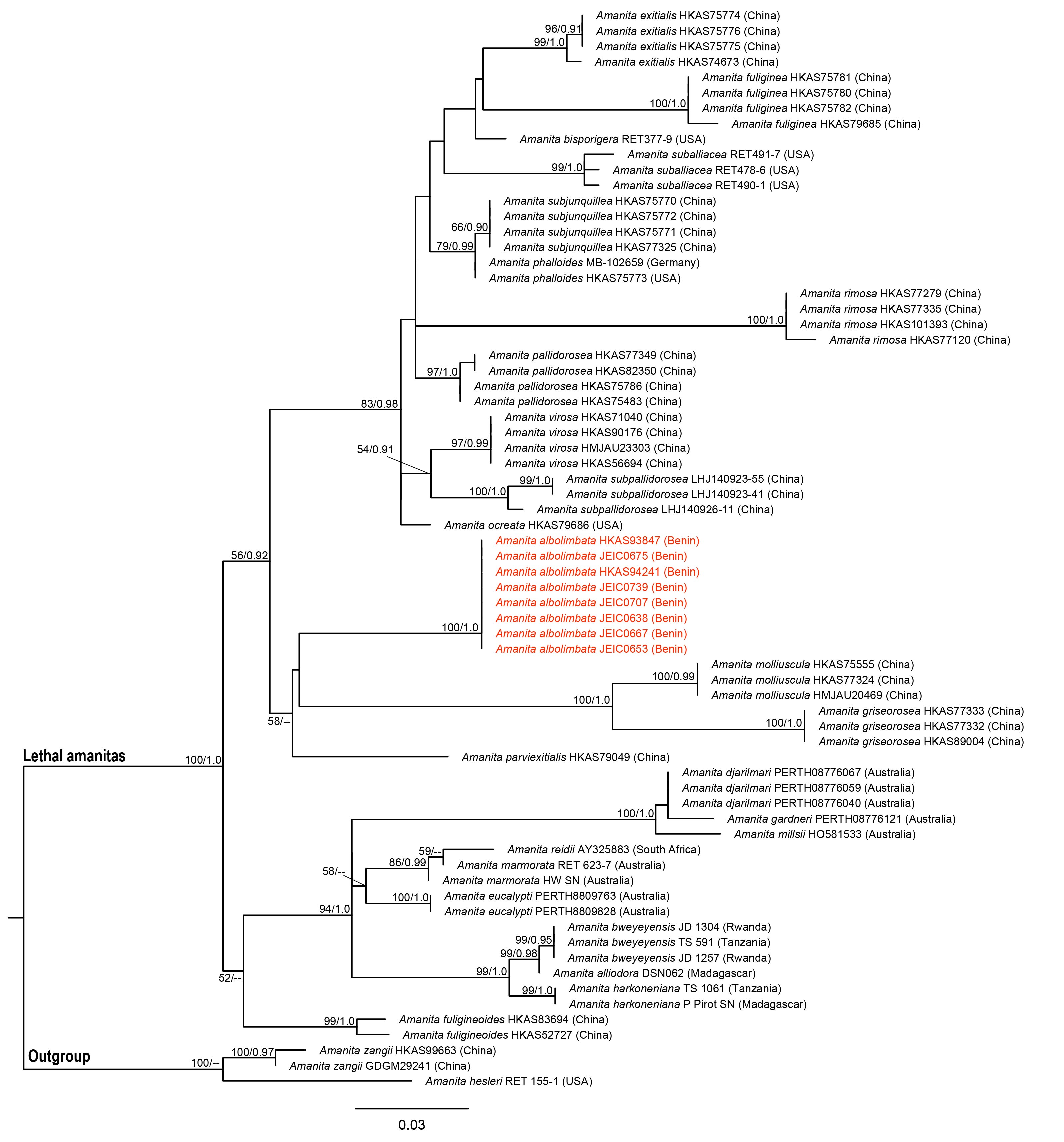

Supplement: Supplementary Figure 4 — Phylogenetic tree inferred by Maximum Likelihood analysis based on nrLSU sequences. Bootstrap values ≥50% and Bayesian posterior probabilities ≥0.90 are reported on branches. Sequences generated in this study are highlighted in red. [file Image_4.JPEG]

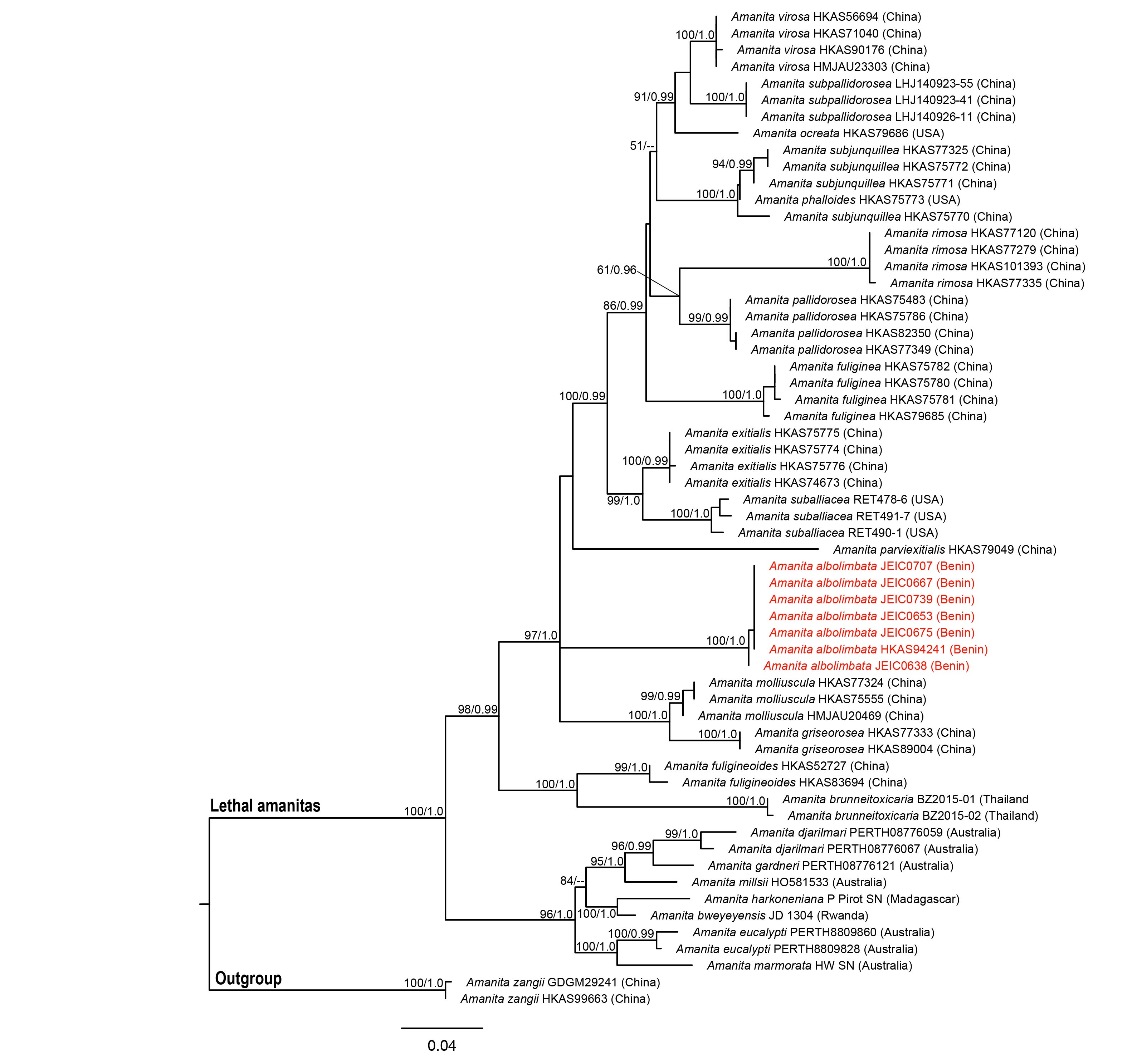

Supplement: Supplementary Figure 5 — Phylogenetic tree inferred by Maximum Likelihood analysis based on rpb2 sequences. Bootstrap values ≥50% and Bayesian posterior probabilities ≥0.90 are reported on branches. Sequences generated in this study are highlighted in red. [file Image_5.JPEG]

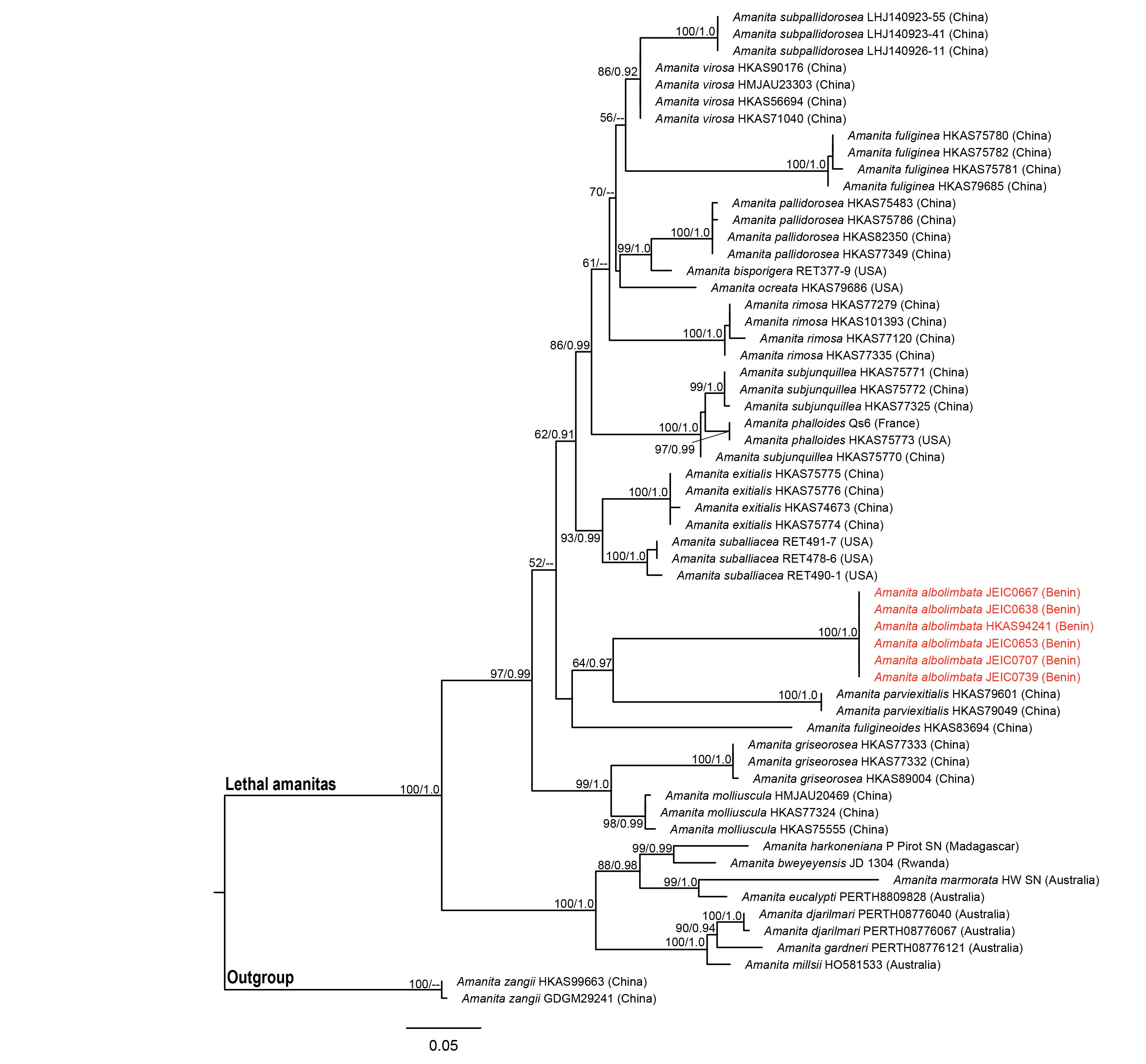

Supplement: Supplementary Figure 6 — Phylogenetic tree inferred by Maximum Likelihood analysis based on tef1-α sequences. Bootstrap values ≥50% and Bayesian posterior probabilities ≥0.90 are reported on branches. Sequences generated in this study are highlighted in red. [file Image_6.JPEG]

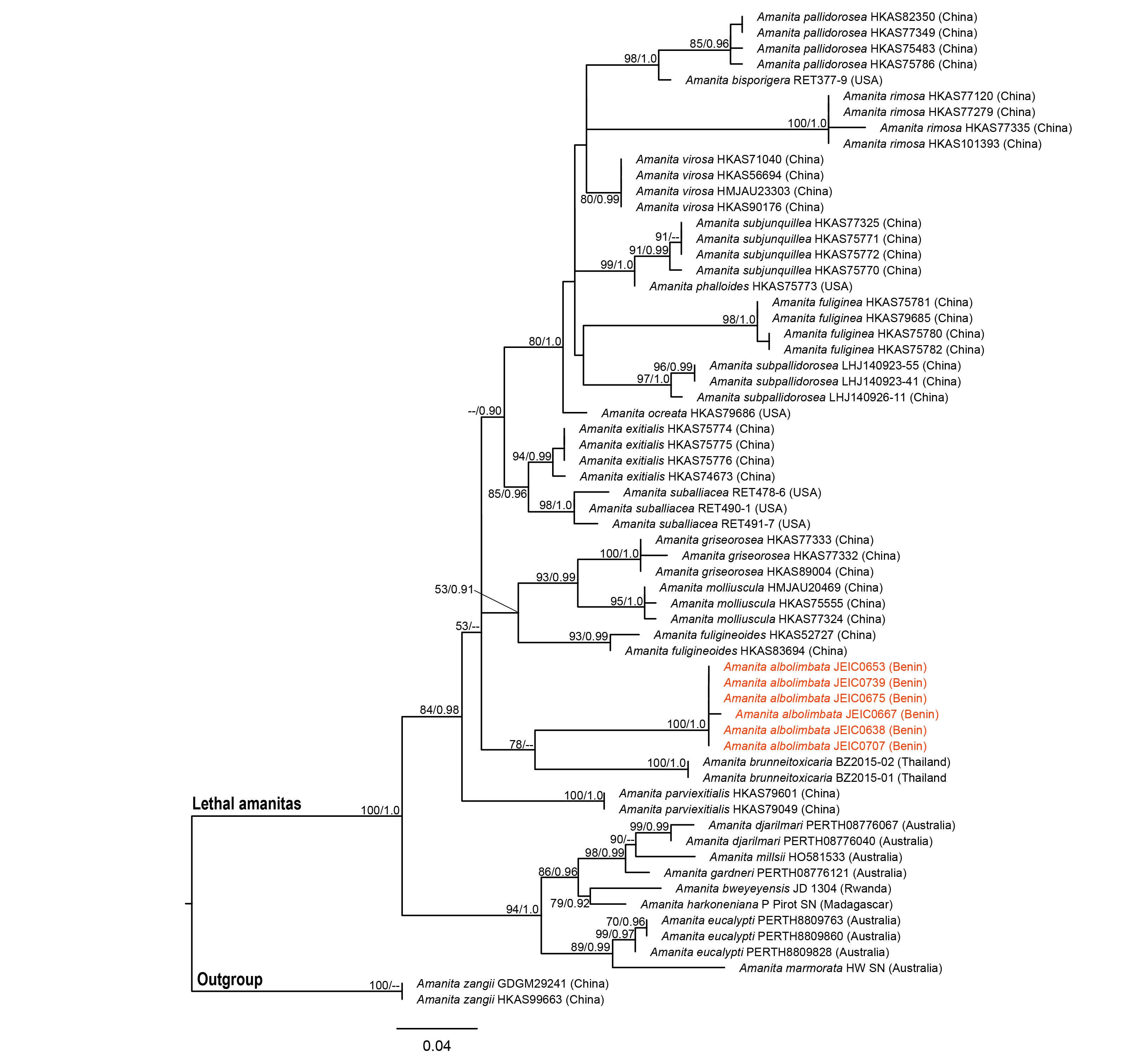

Supplement: Supplementary Figure 7 — Phylogenetic tree inferred by Maximum Likelihood analysis based on β-tubulin sequences. Bootstrap values ≥50% and Bayesian posterior probabilities ≥0.90 are reported on branches. Sequences generated in this study are highlighted in red. [file Image_7.JPEG]
